# The Choice of Resin-Bound Ligand Affects the Structure and Immunogenicity of Column-Purified Human Papillomavirus Type 16 Virus-Like Particles

**DOI:** 10.1371/journal.pone.0035893

**Published:** 2012-04-26

**Authors:** Hyoung Jin Kim, Su Jeung Lim, Hye-Lim Kwag, Hong-Jin Kim

**Affiliations:** College of Pharmacy, Chung-Ang University, Seoul, South Korea; National Cancer Institute, United States of America

## Abstract

Cell growth conditions and purification methods are important in determining biopharmaceutical activity. However, in studies aimed at manufacturing virus-like particles (VLPs) for the purpose of creating a prophylactic vaccine and antigen for human papillomavirus (HPV), the effects of the presence of a resin-bound ligand during purification have never been investigated. In this study, we compared the structural integrity and immunogenicity of two kinds of VLPs derived from HPV type 16 (HPV16 VLPs): one VLP was purified by heparin chromatography (hHPV16 VLP) and the other by cation-exchange chromatography (cHPV16 VLP). The reactivity of anti-HPV16 neutralizing monoclonal antibodies (H16.V5 and H16.E70) towards hHPV16 VLP were significantly higher than the observed cHPV16 VLP reactivities, implying that hHPV16 VLP possesses a greater number of neutralizing epitopes and has a greater potential to elicit anti-HPV16 neutralizing antibodies. After the application of heparin chromatography, HPV16 VLP has a higher affinity for H16.V5 and H16.E70. This result indicates that heparin chromatography is valuable in selecting functional HPV16 VLPs. In regard to VLP immunogenicity, the anti-HPV16 L1 IgG and neutralizing antibody levels elicited by immunizations of mice with hHPV16 VLPs were higher than those elicited by cHPV16 VLP with and without adjuvant. Therefore, the ability of hHPV16 VLP to elicit humoral immune responses was superior to that of cHPV16 VLP. We conclude that the specific chromatographic technique employed for the purification of HPV16 VLPs is an important factor in determining the structural characteristics and immunogenicity of column-purified VLPs.

## Introduction

Of all the types of human papillomavirus (HPV), type 16 is considered to be the most significant, as it is responsible for approximately 50% of cervical cancers [1]. HPV is an epitheliotropic, non-enveloped virus that has a capsid composed of L1 (major) and L2 (minor) proteins [2]. Virus-like particles (VLPs) composed of 72 capsomeres (360 L1 proteins) are a major component of prophylactic HPV vaccines because the VLPs are structurally similar to naturally occurring HPV capsids and display conformation-specific neutralization epitopes [3]. Currently, there are two kinds of VLP-based prophylactic vaccines. One is Gardasil® (Merck), which uses a *Saccharomyces cerevisiae* (*S. cerevisiae*) expression system, and the other is Cervarix™ (GlaxoSmithKline), which uses an insect cell expression system [4].

HPV VLPs have been used as surrogates of the native HPV virion in studies of HPV structure, infection mechanisms and epitope display, as the continuous production of native HPV virion is practically impossible *in vitro* because the production and assembly of native HPV virions are strictly controlled by the cell cycle [2], [5]. In addition, HPV VLPs have been used as antigens in competitive immunoassays aimed at measuring neutralizing antibody titers in vaccine efficacy studies [6].

Numerous viruses, including HPV, undergo conformational changes as they interact with cell surface receptors [7], [8], [9], and these conformational changes influence the selection of immunodominant epitopes on the capsid surface [9]. During HPV infection, L1 protein must first bind to heparan sulfate proteoglycans (HSPGs) present on basement membranes (BM) exposed by wounding [10]. The HPV capsid undergoes a conformational change that exposes the N-terminus of the minor capsid protein L2 when the virus interacts with HSPGs [11], and an exposed N-terminal L2 residue is believed to interact with a secondary receptor. The interaction between heparin and the HPV VLP is thought to result in a VLP conformational change. Selinka *et al.* have suggested that the reactivity of anti-HPV31 L1 monoclonal antibody (Mab) towards HPV31 VLPs prior to and after heparin binding are different [7]. In addition, it has been known that HSPGs interact with correctly folded and intact HPV VLPs, indicating that the use of HSPG as a ligand is important in controlling the quality of HPV VLPs.

In the manufacture of recombinant HPV VLP, the interaction between the VLP and resin-bound ligand during purification has the potential to affect the structure and immunogenicity of the resulting VLP. However, the effect of the resin-bound ligand used in manufacturing HPV VLPs has not been studied until now due to the complexity of the purification process. Previous methods developed for purifying HPV VLPs are not only inefficient but also inconvenient. They require several chromatography steps or ultracentrifugation onto a sucrose cushion followed by size-exclusion chromatography [12], [13], [14]. Such methods are only useful for small-scale purification. Therefore, considerable effort has been made to simplify and improve yields. We have developed two single-step chromatographic methods for purifying HPV16 VLPs produced in *S. cerevisiae* [15]: heparin affinity chromatography using heparin-bounded resin and cation-exchange chromatography using phosphocellulose. These methods are clearly related in that both resins are characterized by a high negative charge density. In the former case, the charge is supplied by the sulfate groups of the heparin, and in the latter case, the charge is supplied by the phosphate groups of the phosphocellulose. As these chromatographic methods are conceptually similar and require the use of a single column type during purification, they offer a unique opportunity to examine the effect of resin-bound ligands on the antigenic activity of the eluted VLPs (Table 1).

**Table 1:** Procedures used to purify the HPV16 VLPs used in this study.

Method	Reference	VLPs	Step	Purification procedure
1	15	hHPV16 VLP	1	Cell disruption
			2	Ammonium sulfate precipitation (take of precipitated protein containing HPV16 VLP)
			3	Removal of precipitated contaminants (take of soluble fraction containing HPV16 VLP)
			4	Heparin chromatography
2	15	cHPV16 VLP	1	Cell disruption
			2	Ammonium sulfate precipitation (take of precipitated protein containing HPV16 VLP)
			3	Removal of precipitated contaminants (take of soluble fraction containing HPV16 VLP)
			4	Cation-exchange chromatography
3	20	scHPV16 VLP	1	Cell disruption
			2	Ultracentrifugation using a sucrose cushion
			3	Size-exclusion chromatography
			4	Cation-exchange chromatography
4	20	schHPV16 VLP^a^	1	Cell disruption
			2	Ultracentrifugation using a sucrose cushion
			3	Size-exclusion chromatography
			4	Cation-exchange chromatography
			5	Heparin chromatography

All the HPV16 VLP preparations were finally dialyzed against 0.325 M NaCl in phosphate buffer pH 7.2.

a The scHPV16 VLP was further separated by heparin chromatography.

In this study, we compared the structural integrity and immunogenicity of HPV16 VLPs purified by heparin chromatography (hHPV16 VLPs) and cation-exchange chromatography (cHPV16 VLPs) (Table 1). Our results indicate that during the purification of HPV VLPs, the resin-bound ligand exerts an important influence on the structural characteristics and immunogenicity of the resulting VLPs.

## Results

### HPV16 VLP Preparations

We prepared four types of HPV16 VLPs to investigate the effect of the purification method on VLP conformation, and we introduced an ammonium sulfate precipitation step to prepare hHPV16 VLP and cHPV16 VLP (Table 1), which is useful for removing contaminating proteins [15]. In addition, the ammonium sulfate treatment increases the extent of intermolecular disulfide bonding, which is critical for the stability of HPV VLPs (Fig. S1) [16]. At the 26^th^ International Papillomavirus Conference, C.B. Buck also suggested that treatment with a low concentration of ammonium sulfate (25 mM) in neutral pH increases the extent of disulfide bonding [17]. In our case, we believe that the ammonium sulfate precipitation step aids in concentrating L1 proteins, and the high concentration allows for a greater chance of establishing of disulfide bonds between L1 proteins. Therefore, the ammonium sulfate precipitation indirectly facilitates disulfide bonding. In addition, this ammonium sulfate precipitation renders the structure of the HPV16 VLP more robust while still removing contaminating proteins. The traditional methods for purifying the HPV VLPs produced in *S. cerevisiae* use successive ultracentrifugation with a sucrose cushion, size-exclusion chromatography and ion-exchange chromatography or microfiltration [12], [18], [19], [20], [21]. They do not employ ammonium sulfate precipitation or a contaminant-removal step. Therefore, we used traditionally purified scHPV16 VLP and resin-purified hHPV16 VLP and cHPV16 VLP to investigate the effect of the resin-bound ligand on VLP conformation (Table 1).

### Comparison of Mab reactivity towards hHPV16 VLP and cHPV16 VLP

**Figure 1:**
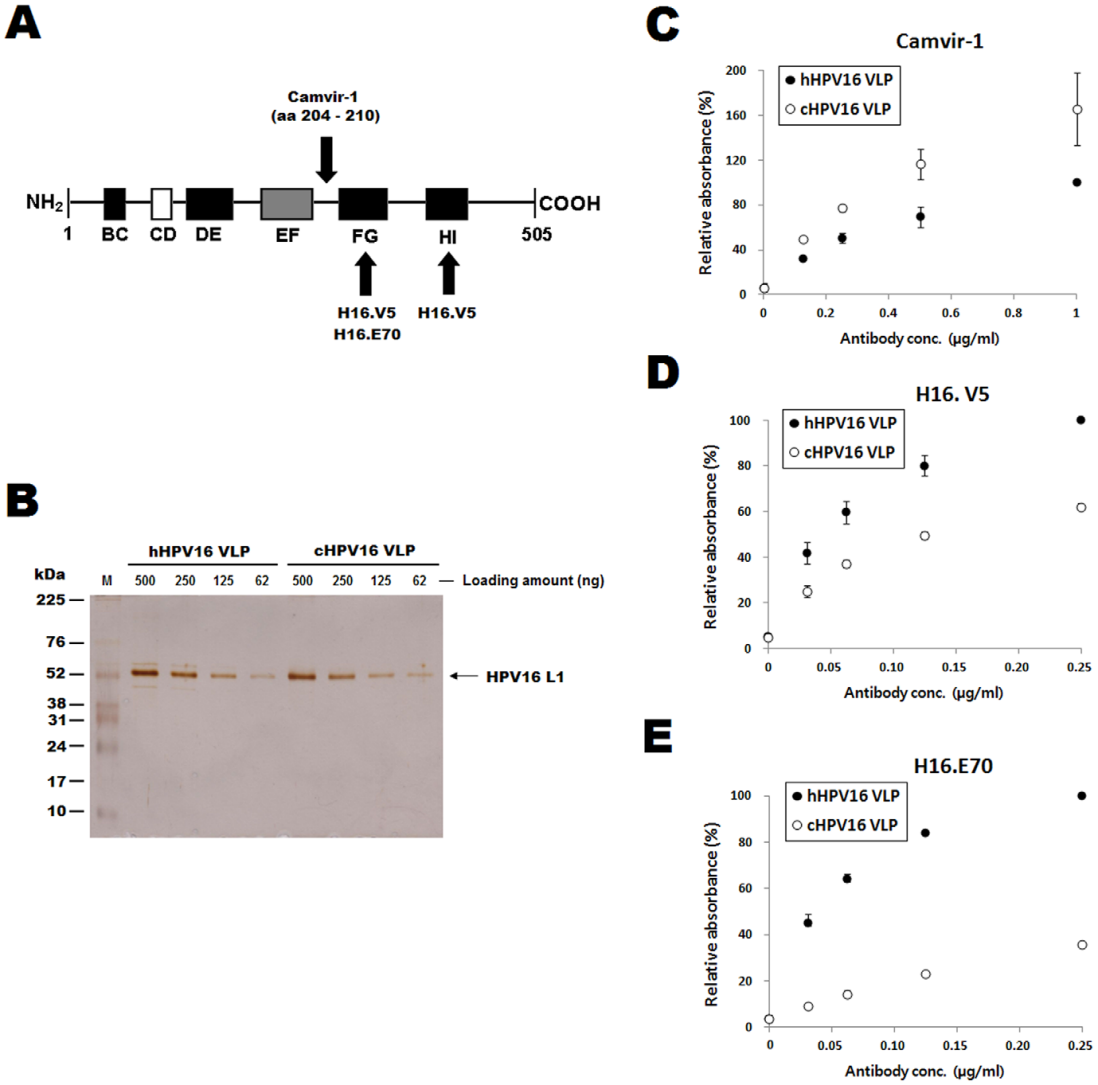
Camvir-1, H16.V5 and H16.E70 reactivity towards hHPV16 VLPs and cHPV16 VLPs. The HPV16 L1 protein residues recognized by H16.V5, H16.E70 and Camvir-1 are displayed graphically in (A). BC, CD, DE, EF, FG and HI indicate the loop structures in HPV16 L1. The numbers refer to the amino acid residues as counted from the N-terminus, the black boxes indicate loops covering the solvent-exposed face of the capsid, and the white box (CD) indicates an internal loop. The gray box (EF) indicates a loop partly located on the outside of the capsid. The hHPV16 VLP and cHPV16 VLP concentrations were confirmed by SDS-PAGE prior to running ELISAs (B). The protein concentration of each VLP preparation was determined by Bradford protein assay, and 500 to 62 ng of proteins were loaded for SDS-PAGE analysis. M indicates the molecular weight marker. Camvir-1, H16.V5 and H16.E70 reactivity towards hHPV16 VLPs and cHPV16 VLPs was determined by direct ELISA. The ELISA results are presented in C, D and E, respectively. The ODs of the hHPV16 VLPs after reaction with 1 µg/ml of Camvir-1, 0.25 µg/ml of H16.V5 and 0.25 µg/ml of H16.E70 were set at 100% in C, D and E, respectively. The ELISA values are the means ± SD of two independent assays.

The HPV16 VLP binding residues for H16.V5 and H16.E70 antibodies are known to be critical for eliciting anti-HPV16 neutralizing antibodies [22]. The H16.V5 and H16.E70 antibodies recognize conformational epitopes on the surface of HPV16 VLPs [23]. H16.V5 recognizes amino acids (aa) 266–297 and 339–365 located in the FG and HI loops, respectively, while H16.E70 recognizes aa 282 in the FG loop (Fig. 1A) [24]. Thus, H16.V5 and H16.E70 recognize different epitopes on the surface of HPV16 VLPs, although there is some overlap in the residues recognized by the two antibodies. The Camvir-1 antibody recognizes a linear epitope that corresponds to residues 204–210 of HPV16 L1 [25], which are located between the EF and FG loops (Fig. 1A). Previous reports [25], [26] predict that aa 204–210 are not exposed on the surface of fully matured HPV VLP. However, a recent study indicated that there was no significant difference between the reactivity of Camvir-1 towards HPV16 capsomeres and HPV16 VLPs [27], indicating that the epitopes for Camvir-1 seem to be exposed on the VLP surface.

To confirm the purity of L1 protein of each VLP preparation, SDS-PAGE was performed (Fig. 1B). There was no difference between band intensities of L1 proteins in hHPV16 VLP and cHPV16 VLP, indicating that two types of VLPs have same L1 purities and these VLPs are almost 100% pure. As shown in Fig. 1C, Camvir-1 reacted with cHPV16 VLP more strongly than it did with hHPV16 VLP. Therefore, it is thought that the linear epitopes for Camvir-1 were more exposed in cHPV16 VLPs than in hHPV16 VLP. In addition, the reactivity of H16.V5 and H16.E70 towards hHPV16 VLPs were significantly higher than those towards cHPV16 VLP (Fig. 1D and E).

### Effect of heparin chromatography on the conformation of HPV16 VLP

**Figure 2:**
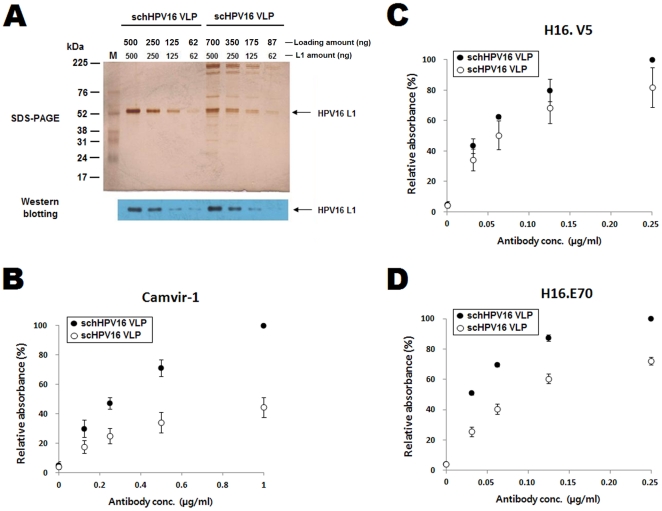
Camvir-1, H16.V5 and H16.E70 reactivity towards scHPV16 VLPs and schHPV16 VLPs. The amounts of L1 proteins contained in scHPV16 VLP and schHPV16 VLP were confirmed by SDS-PAGE and Western blotting prior to performing ELISAs (A). In panel A, loading amount indicates protein amount loaded for SDS-PAGE and Western blot. L1 amount indicates L1 protein amount contained in the loading sample. The L1 protein amount was confirmed by L1 band intensities on SDS-PAGE and Western blot. M indicates the molecular weight marker. The SDS-PAGE and western blot are representatives of duplicate assays. The Camvir-1, H16.V5 and H16.E70 reactivity towards schHPV16 VLPs and scHPV16 VLPs were determined by direct ELISA and are presented in B, C and D, respectively. The ODs of hHPV16 VLPs after reaction with 1 µg/ml of Camvir-1, 0.25 µg/ml of H16.V5 and 0.25 µg/ml of H16.E70 were set at 100% in B, C and D, respectively. The ELISA values are the means ± SD of two independent assays.

To investigate the effect of the heparin chromatography on the conformation of HPV16 VLPs, we compared the Mab reactivity of scHPV16 VLP before and after heparin chromatography (Table 1). The L1 protein concentrations of the two VLP preparations were determined by SDS-PAGE and Western blotting prior to performing ELISAs (Fig. 2A). The proportion of L1 protein of scHPV16 VLP was 70% of total protein while that of schHPV16 VLP was almost 100%. L1 band intensities of scHPV16 VLPs were corresponded with those of schHPV16 VLP when 1.4 times more amounts of scHPV16 VLPs were loaded for the SDS-PAGE and Western blot. For the direct ELISA, therefore, 1.4 times more amounts of protein were coated for scHPV16 VLP. In contrast to the results reported in Fig. 1C, the Camvir-1 reactivity towards schHPV16 VLP was higher than the reactivity towards scHPV16 VLP (Fig. 2B). It is assumed that repeated chromatography resulted in the exposure of the Camvir-1-interacting epitopes on the surface of schHPV16 VLP. At the same time, the H16.V5 and H16.E70 reactivity towards schHPV16 VLP were higher than those towards scHPV16 VLP (Fig. 2C and D).

### Structural integrities of hHPV16 VLPs and cHPV16 VLPs

**Figure 3:**
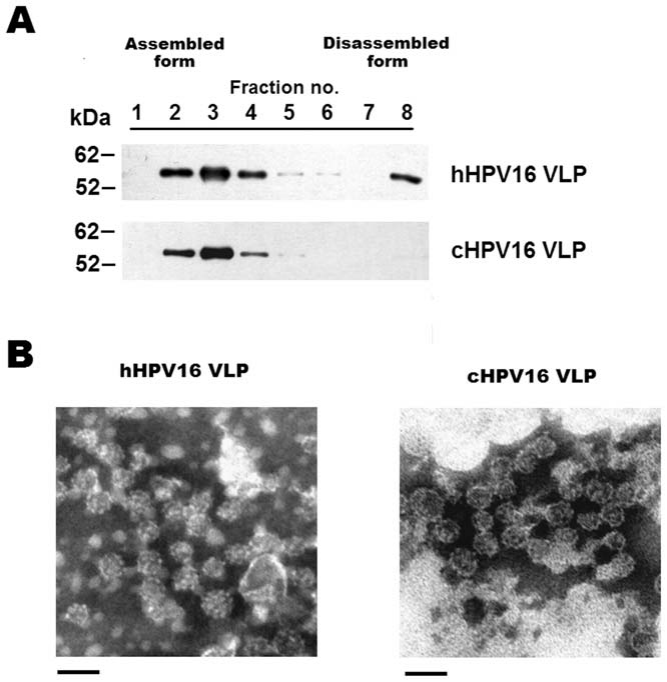
Analysis of the structural characteristics of hHPV16 and cHPV16 VLP. To analyze the structural integrity of the two VLP types, the HPV16 VLPs were fractionated on Optiprep density gradients (A). Eight fractions were collected in experiment A (0.5 ml each). The results of TEM analysis are presented in panel B. Magnification is 41,000× (bars 50 nm).

To further investigate the structural integrity of the hHPV16 VLP and cHPV16 VLP, we used density gradient ultracentrifugation and transmission electron microscopy (TEM) analysis, both of which have been previously used to analyze capsid structure [16], [28], [29]. The hHPV16 VLP and cHPV16 VLP were subjected to analytical centrifugation in Optiprep density gradients (Fig. 3A). All cHPV16 VLPs were recovered in large particles, while the hHPV16 VLPs appeared to have been partially dissociated (Fig. 3A). In the TEM analysis, two types of VLPs have sizes ranging from 25 to 45 nm, and there was no significant difference in the shape of the two VLP preparations (Fig. 3B).

**Figure 4:**
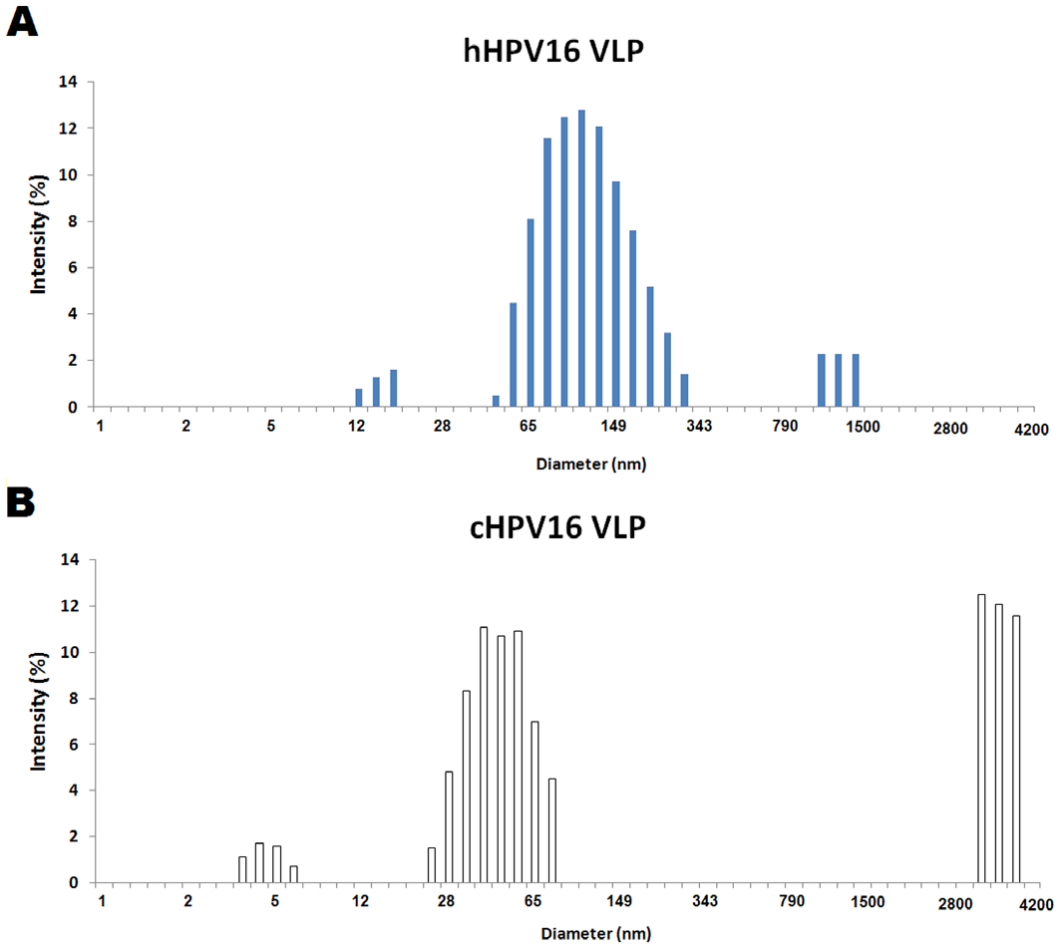
Particle size distributions of hHPV16 VLP and cHPV16 VLP. The HPV16 VLP populations along with their associated hydrodynamic diameters were analyzed by DLS as described in the Materials and Methods section. Each HPV16 VLP was prepared in 25 mM MOPS containing 75 mM NaCl pH 7.0 and adjusted to 40 µg/ml. Panel A and B are representatives of duplicate measurements of the hHPV16 VLP and cHPV16 VLP populations, respectively.

We further investigated the particle size distributions of two kinds of HPV16 VLPs using dynamic light scattering (DLS). As shown in Fig. 4, most hHPV16 VLPs were confirmed to have sizes ranging from 60 to 160 nm while the cHPV16 VLPs had two different populations with sizes ranging from 40 to 100 nm and 2800 to 4200 nm, respectively. The mean hydrodynamic diameters of the hHPV16 VLPs and cHPV16 VLPs were confirmed to be 102 and 214 nm, respectively. In addition, it was confirmed that both hHPV16 and cHPV16 VLPs have small sizes of particles less than 25 nm. These results suggest that not only VLPs but also capsomeres and L1 aggregates may be purified by two types of purification methods.

Unlike the results of TEM, the sizes of VLPs were significantly larger than 50 nm in DLS analysis. DLS shows how a particle diffuses within a fluid while TEM just shows the shape and size of each VLP particle. Therefore, it seems that HPV16 VLPs form VLP masses or incorrectly folded L1s form aggregates when they diffuse in solution. In summary, Fig. 4 implies that the particle sizes of hHPV16 VLP are significantly different from those of cHPV16 VLP in solution and cHPV16 VLPs have more L1 aggregates than hHPV16 VLPs. Increasing the hydrodynamic diameter of HPV VLP is significantly correlated with decreasing *in vitro* antigenicity [30]. Indeed, the *in vitro* antigenicity of the cHPV16 VLP was confirmed to be lower than that of the hHPV16 VLP (Fig. 1). Therefore, these results re-confirm that the structural integrity of hHPV16 VLP is significantly different from that of cHPV16 VLP.

### Humoral immune responses following immunization with hHPV16 VLPs and cHPV16 VLPs

We compared the lipopolysaccharide (LPS) contents of hHPV16 and cHPV16 VLP prior to immunization. The hHPV16 and cHPV16 VLP concentrations were 0.49 and 0.32 EU per 1000 ng L1 protein, respectively (Table S1). It has been clearly demonstrated that these LPS levels do not affect humoral and cellular immune responses in mice [31]. Additionally, it was confirmed that the two HPV16 VLP preparations do not stimulate immune cells in polymyxin B inhibition tests (Fig. S2). All HPV16 VLP preparations were dialyzed against 0.325 M NaCl in phosphate buffer pH 7.2 plus 0.01% Tween 80 prior to the immunizations.

**Figure 5:**
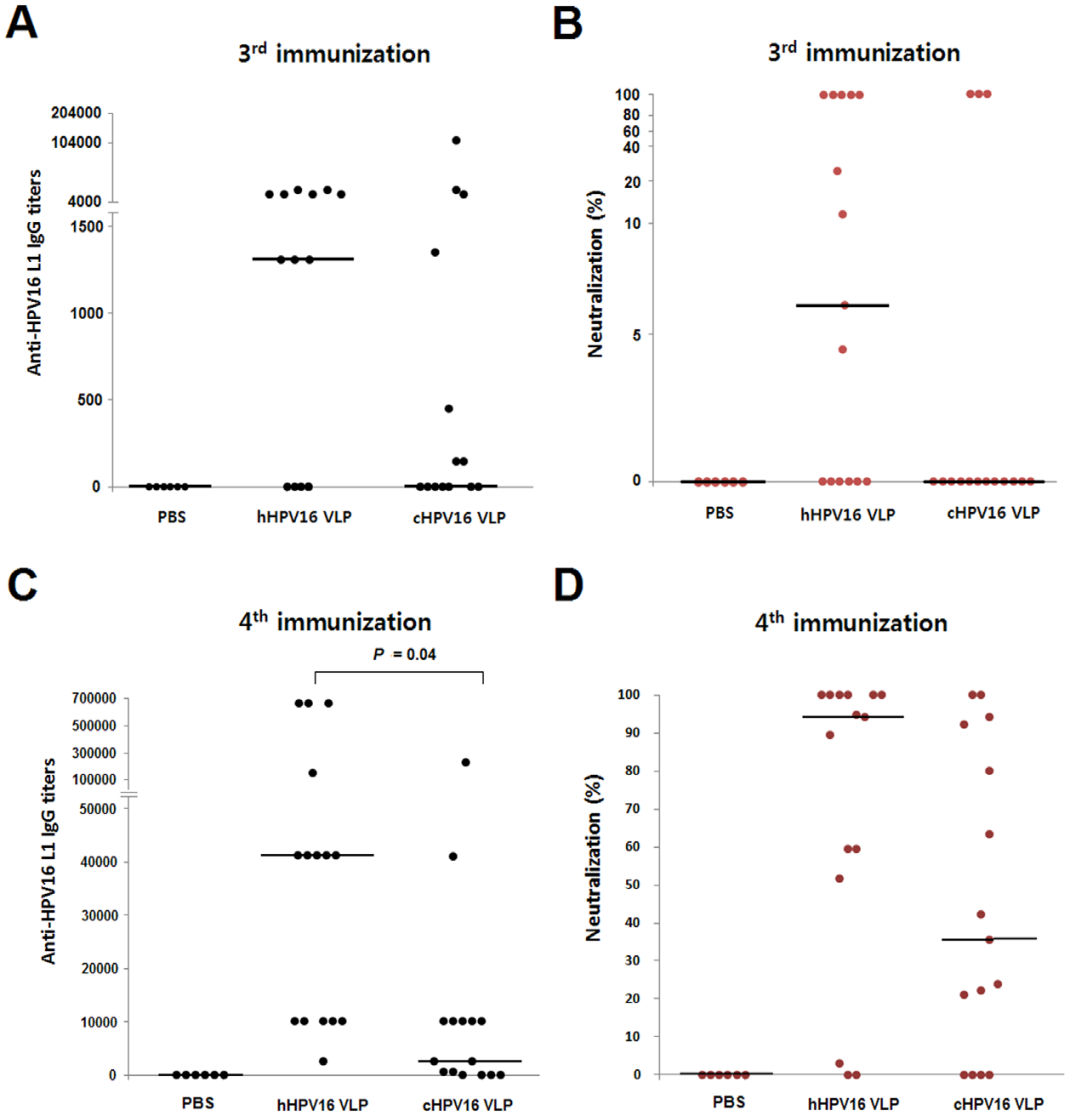
Immunization Protocol-1 Anti-HPV16 L1 IgG titer and neutralization activity resulting. Mice were immunized according to immunization protocol-1 (Table 2). The mice were immunized four times with 100 µl of PBS, 8 ng of hHPV16 VLP or 8 ng of cHPV16 VLP at two-week intervals, in the absence of adjuvant. Ten days after the 3^rd^ and 4^th^ immunization, the mice sera were obtained and analyzed. Panels A and B present the anti-HPV16 L1 IgG titer and neutralization activity after the 3^rd^ immunization, respectively. Panels C and D present the anti-HPV16 L1 IgG titer and neutralization activity after the 4^th^ immunization, respectively. The horizontal bars are median values of the IgG titer and neutralization activity (PBS, n = 6; hHPV16 VLP, n = 15; cHPV16 VLP, n = 15).

**Figure 6:**
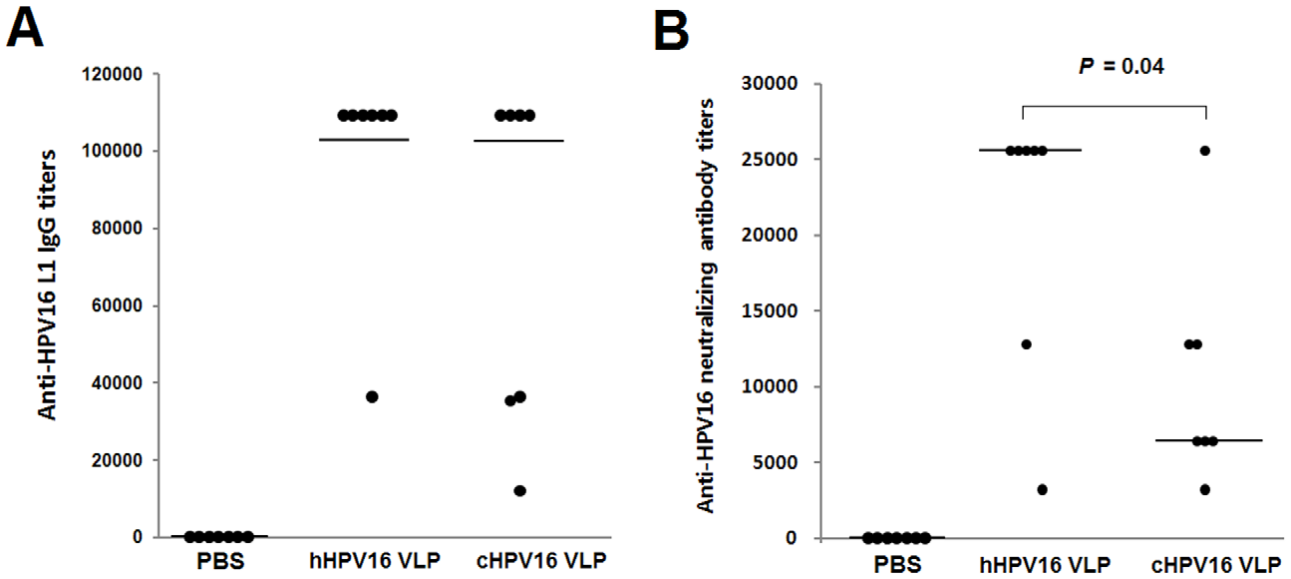
Immunization Protocol-2 Anti-HPV16 L1 IgG and neutralization antibody titers. The anti-HPV16 L1 IgG and anti-HPV16 neutralizing antibody titers of the PBS-, hHPV16 VLP- and cHPV16 VLP-immunization groups are presented in panels A and B, respectively. For the immunizations, the mice were subcutaneously injected three times with 100 µl of PBS, 1000 ng of hHPV16 VLP or 1000 ng of cHPV16 VLP at two-week intervals (protocol-2 in Table 2). The anti-HPV16 L1 IgG and anti-HPV16 neutralizing antibody titers were determined by ELISA and SEAP-based neutralization assays, respectively. The data values of seven individual mice (n = 7) are represented with dots. The horizontal bars indicate the median titers of the anti-HPV16 L1 IgG and neutralization antibodies.

We investigated the immunogenicity of two kinds of HPV16 VLPs through three separate protocols referred to as protocol-1, 2 and 3 (Table 2). In protocol-1, the mice were immunized four times with 8 ng of hHPV16 VLP or cHPV16 VLP without adjuvant. As shown in Fig. 5, the immunization of hHPV16 VLP elicited anti-HPV16 L1 IgG and neutralizing antibody more strongly than immunization with cHPV16 VLP. In protocol-2, the mice were immunized three times with 1000 ng of hHPV16 VLP or cHPV16 VLP, in combination with the adjuvant aluminum hydroxide, to investigate prophylactic vaccine potential (Fig. 6A and B). Immunization with hHPV16 VLP elicited anti-HPV16 neutralizing antibody more strongly than immunization with cHPV16 VLP, but there was no significant difference in the anti-HPV16 L1 IgG titers (Fig. 6 and Fig. S3). Additionally, the immunogenicity of schHPV16 VLP was also confirmed to be higher than that of scHPV16 VLP (Fig. S4). Therefore, HPV16 VLPs, which have higher affinities for H16.V5 and H16.E70 and smaller mean hydrodynamic diameter, also demonstrated higher general immunogenicity (Fig. 1, 2, 4, 5 and 6 and Fig. S4).

**Table 2:** Mouse immunization protocols used in this study.

Protocol	Immunization route	Dosing amount	Immunization schedule	Adjuvant	Blooding	Spleen isolation	Results
1	Subcutaneous	8 ng per dose	Four times at two-week intervals	None	Ten days after 3^rd^ and 4^th^ immunizations	—	Figure 5
Figure S4							
2	Subcutaneous	1000 ng per dose	Three times at two-week intervals	Aluminum hydroxide (200 µg per dose)	Ten days after 3^rd^ immunization	—	Figure 6
Figure S3							
3	Subcutaneous	1000 ng per dose	Two times at a two-week interval	Aluminum hydroxide (200 µg per dose)	—	Ten days after 2^nd^ immunization	Figure 7

### Proliferative responses of CD3^+^ T lymphocytes following immunization with hHPV16 VLPs and cHPV16 VLPs

**Figure 7:**
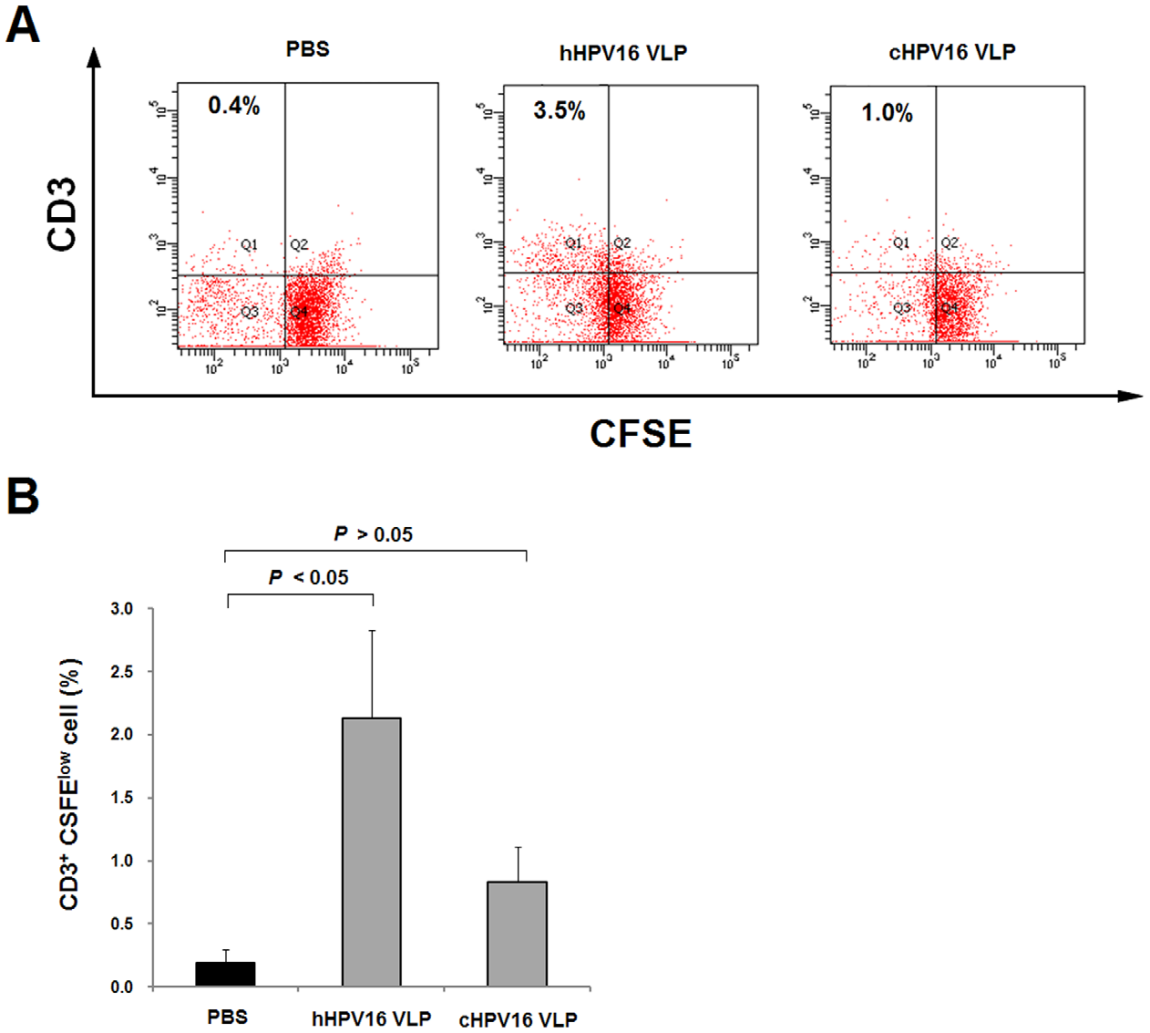
*In vitro* CD3^+^ cell proliferation measured using CFSE labeling. To measure HPV16 VLP-specific CD3^+^ cell proliferation, mice were immunized two times with 1000 ng of hHPV16 VLP or cHPV16 VLP at a two-week interval (protocol-3 in Table 2). The PBS-immunization group received 100 µl of PBS using same protocol described above. Ten days after the last immunization, mice spleens were isolated and labeled with CFSE. The CFSE-labeled spleen cells were re-stimulated with 10 µg/ml of HPV16 VLP (hHPV16 VLP and cHPV16 VLP in a 1∶1 ratio) and cultured for 5 days. CD3^+^ cells were detected using anti-mouse CD3 PerCP-eFluor® 710 antibodies. Panel A is representative the flow cytometry results of three individual mice demonstrate the proliferation of CD3^+^ total live lymphocyte cells. To score proliferated CD3^+^ cells, total live lymphocytes were gated from forward and side scatter, and the upper-left segment of each graph was counted using FITC and PerCP eFluor scatter. Panels B presents the mean ± SEM (n = 3).

CD3 is a well-characterized marker of T lymphocytes [32], [33]. To investigate HPV16 VLP-specific proliferative responses, mice were immunized two times with 1000 ng per mouse of hHPV16 and cHPV16 VLPs (protocol-3, Table 2). Ten days after the last immunization, mice splenocytes were isolated and re-stimulated with HPV16 VLP, and the number of proliferated CD3^+^ cells was scored. As shown in Fig. 7A and B, the proliferative response of CD3^+^ T cells in the hHPV16 VLP-immunization group was significantly higher than the response observed in the cHPV16 VLP-immunization group. These results indicate that immunogenicity of hHPV16 VLP in eliciting humoral and cellular immune responses is superior to that of cHPV16 VLP.

## Discussion

In this study, we compared the structural integrity and immunogenicity of hHPV16 VLP and cHPV16 VLP. Our results indicate that the use of heparin chromatography is more advantageous in obtaining highly immunogenic HPV16 VLP when compared to cation-exchange chromatography. The epitopes of H16.V5 and H16.E70 are critical for inducing anti-HPV16 neutralizing antibodies [22], [34], [35]. We found that the affinities of hHPV16 VLP for H16.V5 and H16.E70 were significantly higher than those of cHPV16 VLP and mean hydrodynamic diameter of hHPV16 VLP was smaller than that of cHPV16 VLP. These results suggest that hHPV16 VLPs may have more neutralizing epitopes and superior antigenicity. Indeed, the structural integrity of hHPV16 VLP was reflected by the elicitation of anti-HPV16 neutralizing antibodies in mouse immunization experiment (Fig. 5 and 6). Therefore, we believed that the reactivity of H16.V5 and H16.E70 towards HPV16 VLPs and hydrodynamic diameter of HPV16 VLP can be ideal indicators in predicting the immunogenicity of HPV16 VLPs. Further studies for correlation between the Mab reactivity towards HPV16 VLP and immunogenicity of HPV16 VLP or hydrodynamic diameter of HPV16 VLP and immunogenicity of HPV16 VLP will provide ideas for controlling quality of HPV VLP.

In this study, we separated two forms of hHPV16 VLP by analytical ultracentrifugation: a fully assembled and a dissociated form (Fig. 3A). A TEM analysis indicated that the hHPV16 VLPs appear to be fully assembled (Fig. 3B), but the lack of a monomer band in non-reducing Western blots of hHPV16 VLPs points to the existence of a dissociated form (data not shown). Moreover, it was confirmed most hHPV16 VLPs have sizes between 60 and 160 nm, indicating that those are assembled forms or L1 aggregates (Fig. 4A). Therefore, we assume that the hHPV16 VLP is partially dissociated by hydrostatic pressure during ultracentrifugation (Fig. 3A).

Previously, we confirmed that the final yields of L1 protein resulting from heparin and cation-exchange chromatography are similar [15]. In this study, however, we found that the structure and immunogenicity of the two kinds of HPV16 VLPs were different. Moreover, cHPV16 VLPs were confirmed to contain more L1 aggregates while hHPV16 VLP did not (Fig. 4). These results indicate that the heparin-bound resin more efficiently selects for the correctly assembled HPV16 VLP than phosphate-bound cation exchange resin.

The shielding of key epitopes within the three-dimensional structure of the native envelope trimer of human immunodeficiency virus (HIV) is a major problem in neutralizing vaccine development because the conformational masking offers the virus an opportunity to evade host defenses [36], [37]. This conformational camouflage has also been observed in the case of human parvovirus (HP) B19. HP B19 exposes neutralization epitopes and can be neutralized by neutralizing antibody only after it binds to its receptor on the cell surface [38]. In recent years, the VLP has been used as a platform to display heterogeneous epitopes [39], [40]. Therefore, developing strategies to control epitope display is a high priority in the vaccine field. Our results indicate that the choice of resin-bound ligand during downstream processing affects the displays of epitopes on the surface of virus capsids. We anticipate that further study of resin-bound ligands and virus capsids will provide additional information to aid in efforts to control surface epitope display.

Previously, it has been suggested that the upstream and downstream processing of virus-like particles during production may affect to their structure and immunogenicity [41]. However, there have been considerable limits on the methods used to determine the best chromatography system to use during purification [42], [43] due to a dearth of information how the choice of resin-bound ligand affects the function of the final recovered VLP product. Moreover, until now, there was no study examining how structure and immunogenicity can be directly affected by the manufacturing process. In summary, the results of our study indicate that differences in manufacturing processes can result in functional differences in purified HPV VLP. The resin-bound ligand used in purifying HPV VLPs is an important determinant of the quality and characteristics of the resulting HPV VLP preparation.

## Materials and Methods

### Ethics

Five- to six-week-old female BALB/c mice were purchased from Orient Bio (Orient Bio, South Korea) and acclimatized for 1 week prior to immunization. All animal experiments were performed in accordance with the National Research Council's Guidelines for the Care and Use of Laboratory Animals and with the Chung-Ang University Guidelines for Animal Experiments. In addition, all experiments were approved by the University Committee for animal experiments (approval no. 10-1017).

### Expression and purification of HPV VLPs

HPV16 VLPs were expressed in HPV16 VLP-producing *S. cerevisiae* [44]. The detailed purification procedure used for each HPV16 VLP preparation is presented in Table 1. The hHPV16 and cHPV16 VLPs were purified as previously described [15], and the HPV16 VLP was purified by size-exclusion and cation-exchange chromatography (scHPV16 VLP) as previously described [12], [20]. To compare the conformations of the HPV16 VLPs before and after heparin chromatography, pre-heparin chromatography scHPV16 VLP was used. This HPV16 VLP was further separated by heparin chromatography and designated schHPV16 VLP (Table 1). All HPV16 VLP preparations were finally dialyzed against 0.325 M NaCl in phosphate buffer pH 7.2 plus 0.01% Tween 80 prior to performing experiments.

### Determination of protein concentration

Protein concentration of each VLP preparation was determined with a Bio-Rad Bradford protein assay kit (Bio-Rad Laboratories, USA) with bovine serum albumin (BSA; Pierce, USA) as standard. The purities of L1 proteins were further determined by SDS-PAGE and Western blot analysis described below.

### Direct ELISA using Mabs against HPV16 L1

ELISAs were performed as previously described [27] to investigate the reactivity of Mabs towards HPV16 VLPs. H16.V5 and H16.E70 Mabs were kindly provided by Dr. N. D. Christensen (Pennsylvania State University, College of Medicine USA). Camvir-1 was purchased from Chemicon (Chemicon, USA). A 96-well ELISA plate (Greiner Bio1one, Germany) was coated overnight at 4°C with 400 ng of HPV16 VLP per well. The plate was blocked with 5% skim milk in PBS plus 0.05% Tween 20 (PBST), and then each of the three Mabs was serially diluted and incubated for 1 h at 37°C. The bound Mabs were detected using HRP-conjugated goat anti-mouse IgG polyclonal antibody (diluted 1∶5000, Bethyl Laboratories, USA). The samples were colorimetrically developed with o-phenylenediamine (Sigma, USA) and measured at an absorbance of 492 nm.

### SDS-PAGE and Western blot analysis

SDS–PAGE was performed using the Laemmli method [45]. Western blot analysis was performed as previously described [20]. The band corresponding to L1 protein was detected using rabbit anti-HPV16 L1 polyclonal antibody together with anti-rabbit IgG antibody (Pierece, USA). The rabbit anti-HPV16 L1 Pab was kindly provided by Dr. J.T. Schiller (NIH, Bethesda, USA). All samples were fractionated using 12% polyacrylamide gels.

### Optiprep density gradients

Optiprep density gradient analysis was performed as previously described [46]. Beckman polyallomer tubes (Beckman Coulter, Fullerton, CA) were successively packed from the bottom of the tube with 39, 33 and 27% iodixanol (Sigma, USA) in phosphate-buffered saline (PBS) containing 0.8 M NaCl and purified HPV16 VLP. The preparations were centrifuged at 234,000 g for 4 h. Next, 500-ul fractions were collected in siliconized microcentrifuge tubes (Sigma, USA) by puncturing the bottom of the tube, and HPV16 L1 proteins were detected using SDS-PAGE and western blotting as described above.

### Transmission electron microscopy (TEM)

For TEM analysis, purified hHPV16 VLP and cHPV16 VLP were prepared and analyzed as previously described [44].

### Dynamic light scattering (DLS)

The size distribution of each HPV16 VLP was analyzed using a DLS-700 system (Otsuka Electronics, Japan) at 23°C. Each HPV16 VLP was prepared in 25 mM MOPS containing 75 mM NaCl pH 7.0 (final conc. of L1s were adjusted to 40 µg/ml). The preparations were measured using duplicate assays, and the representative result of each VLP preparation was presented.

### Mice Immunization

Three kinds of immunization protocols were used to investigating the immunogenicity of the HPV VLPs. The protocols are briefly summarized in Table 2. In immunization protocol-1, six-week-old female mice were divided into three groups, each consisting of 6 or 8 mice. A control group was given PBS, and the hHPV16- and cHPV16 VLP-immunization groups received subcutaneously injections of 8 ng of hHPV16 VLP or cHPV16 VLP without adjuvant. The three groups were immunized four times at two-week intervals (Table 2). In immunization protocol-2, a control group was given PBS in combination with aluminum hydroxide (200 µg per dose) [47]. The hHPV16 VLP- and cHPV16 VLP-immunization groups each received subcutaneously injections of 1000 ng of hHPV16 VLPs or cHPV16 VLP, in combination with aluminum hydroxide (200 µg per dose). The three groups were immunized three times at two-week intervals (Table 2). In protocol-3, to investigate the lymphoproliferative responses elicited by immunizations with the two kinds of HPV16 VLPs, the mice were immunized two times at two-week intervals with 1000 ng of hHPV16 VLPs or cHPV16 VLP, in combination with aluminum hydroxide (200 µg per dose, Table 2). The immunization doses were based on a previous report [27]. Ten days after the last boost injection, splenocytes were obtained from each group for flow cytometry analysis. Sera were obtained by centrifugation of whole blood, and stored at −70°C until analysis.

### Carboxyfluorescein succinimidyl ester-based splenocyte proliferation assay

Splenocytes from the PBS-, hHPV16 VLP- and cHPV16 VLP-immunized mice were labeled with carboxyfluorescein succinimidyl ester (CFSE) using a CellTrace™ CFSE cell proliferation kit (Invitrogen, USA). The CFSE-labeled splenocytes were cultured in 96-well flat-bottom cell culture plates (1.2×10^6^ cells/well) for 5 day at 37°C with 10 µg/ml of HPV16 VLP (hHPV16 VLP and cHPV16 VLP mixed in a 1∶1 ratio). The cells were harvested, washed with staining buffer (1% FBS in PBS) and stained with anti-mouse CD3 peridinin chlorophyll protein-eFluor® 710 (PerCP-eFluor®) antibody (eBioscience, USA). The cells were washed twice with the staining buffer, and the stained cells were examining with a FACSAria flow cytometer (BD Bioscience, USA). Thirty thousand events were acquired with the live lymphocyte gates during the scoring of proliferated CD3^+^ CFSE^low^ cells.

### Titration of anti-HPV16 L1 IgG

The titers of anti-HPV16 L1 IgG in the mouse sera were determined by indirect ELISA as previously described [48]. Briefly, 96-well ELISA plates were coated and incubated overnight with 100 ng of cHPV16 VLP per well at 4°C and blocked with 2% BSA in PBST. Serial three-fold dilutions of serum were added to the wells and incubated for 1 h at 37°C. Next, HRP-conjugated goat anti-mouse IgG was added and the plates were incubated for 1 h at 37°C. The color reaction was developed as described in the direct ELISA section above. End-point titers were established at an OD of 2 times the OD of the control serum [12].

### Neutralization assay

To investigate the neutralizing ability of anti-HPV16 L1 mouse sera, a pseudovirus (PsV)-based neutralization assay was performed as previously described [49]. The plasmids p16sheLL (containing both HPV16 L1 and L2 gene) and pYSEAP (reporter plasmid) were kindly provided by Dr. J.T. Schiller (NIH, Bethesda, USA). For the neutralization assay, 293TT cells were seeded in 96-well tissue culture plates at a density of 3×10^4^ cells/well and incubated for 4 h at 37°C. Optiprep density gradient-purified PsV stock was diluted 400-fold and incubated with dilutions of the sera at 4°C for 1 h. Next, the PsV-serum mixtures were added to the seeded cells and further incubated for 72 h at 37°C. The secreted alkaline phosphate (SEAP) activity of the culture supernatants was measured by a colorimetric SEAP assay. The neutralization activities of the mice sera obtained from immunization protocol-1 were determined as described previously with slight modification [27]. The equation is as follows: neutralization (%) = (value for PsV alone−value for PsV mice serum)/(value for PsV alone−value for blank)×100. The neutralization antibody titers of the mice sera obtained from immunization protocol-2 were determined as the reciprocal of the highest dilution that caused at least a 50% reduction in SEAP activity when compared to controls treated with PsV alone.

### Statistical analysis

The statistical significance of differences between groups was determined using two-tailed Student's t-tests. P<0.05 indicated a statistically significant difference.

## Supporting Information

Figure S1 **Effect of ammonium sulfate precipitation on the extension of intermolecular disulfide bonding of HPV16 VLP.** To compare the degrees of intermolecular disulfide bonding between HPV16 VLPs before and after ammonium sulfate precipitation, non-reducing Western blotting was performed as described at the website of National Cancer Institute (NCI) (http://home.ccr.cancer.gov/Lco/ImprovedMaturation.htm). To detect HPV16 L1 protein, rabbit anti-HPV16 L1 polyclonal antibody and HRP-conjugated goat anti-rabbit IgG was used.
(TIF)

Figure S2 **Measurements of endotoxin levels contained in hHPV16 VLP and cHPV16 VLP.** To measure the endotoxin levels, RAW264.7 (2×10^4^ cells / well) cells were seeded in a 96-well cell culture plate 24 hours prior to stimulations. To block Toll-like receptor 4 on RAW264.7 cells, the cells were treated with 50 µg/ml of polymyxin B (Sigma, USA) for 30 min at 37°C prior to LPS and HPV VLPs treatments. The cells were treated with LPS, hHPV16 VLP and cHPV16 VLP at concentrations of 10 ng/ml, 10 µg/ml and 10 µg/ml, respectively. Four hours after the treatments, the levels of TNF-α in the culture supernatants was measured using an ELISA kit according to manufacturer's instructions (BD Bioscience, USA). Values are presented as the means ± SD of duplicate assays.
(TIF)

Figure S3 **Neutralization assay results.** The mice were immunized three times with 1000 ng of hHPV16 VLP or cHPV16 VLP, in combination with aluminum hydroxide (protocol-2, Table 2). The mice sera were serially diluted and incubated with Optiprep density gradient-purified HPV16 PsVs for 1 h at 4°C. The PsV and mice sera mixtures were added to pre-plated 293TT cells and cultured for 72 h at 37°C. The secreted SEAP of each well was developed using a 4-nitrophenly phosphate disodium salt hexahydrate (Sigma, USA). B and P of the figure captions indicate the ells cultured with media only (blank) and PsV only, respectively.
(TIF)

Figure S4 **Anti-HPV16 L1 IgG titers and neutralization activities of mice sera following immunizations with schHPV16 and scHPV16 VLP.** Mice were immunized subcutaneously three times with 8 ng of schHPV16 VLP or scHPV16 VLP without adjuvant. Ten days after the last immunization, the sera were obtained and analyzed as described in the Materials and Methods to determine the anti-HPV16 L1 IgG titers and neutralization activities. The horizontal bars are the median values (PBS, n = 6; schHPV16 VLP, n = 8; scHPV16 VLP, n = 8).
(TIF)

Table S1 **Determinations of LPS levels in Mock and HPV16 VLPs.** Mock samples were prepared form the cell lysate of parent cell of HPV16 L1-producing *S. cerevisiae*. The concentrations of hHPV16 VLP and cHPV16 VLP were determined by SDS-PAGE and Western blotting prior to the assay. The LPS level of each sample was determined using the limulus amoebocyte lysate (LAL) based colorimetric system (ToxinSensor™, GenScript, USA) according to the manufacture's instruction.
(DOCX)
