# Supplementary figures and images for: The Choice of Resin-Bound Ligand Affects the Structure and Immunogenicity of Column-Purified Human Papillomavirus Type 16 Virus-Like Particles
Source: PLoS One. 2012 Apr 26;7(4):e35893. doi: 10.1371/journal.pone.0035893 (PMC3338541; doi:10.1371/journal.pone.0035893)

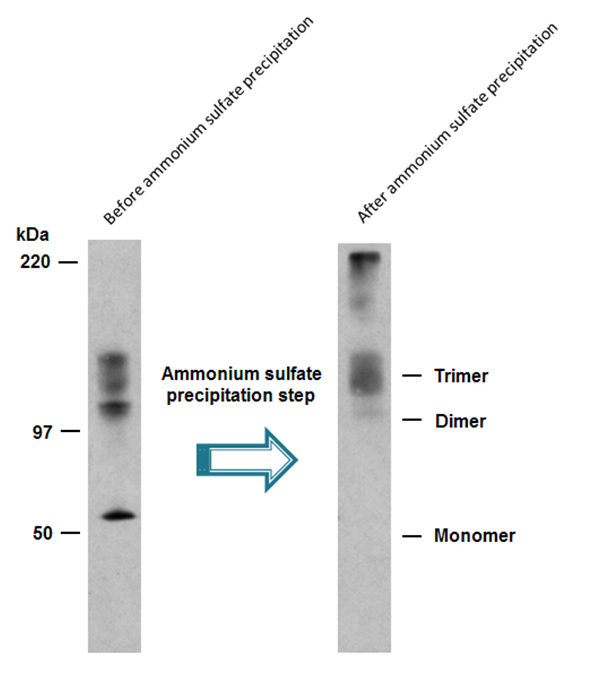

Supplement: Figure S1 — Effect of ammonium sulfate precipitation on the extension of intermolecular disulfide bonding of HPV16 VLP. To compare the degrees of intermolecular disulfide bonding between HPV16 VLPs before and after ammonium sulfate precipitation, non-reducing Western blotting was performed as described at the website of National Cancer Institute (NCI) (http://home.ccr.cancer.gov/Lco/ImprovedMaturation.htm). To detect HPV16 L1 protein, rabbit anti-HPV16 L1 polyclonal antibody and HRP-conjugated goat anti-rabbit IgG was used. (TIF) [file pone.0035893.s001.tif]

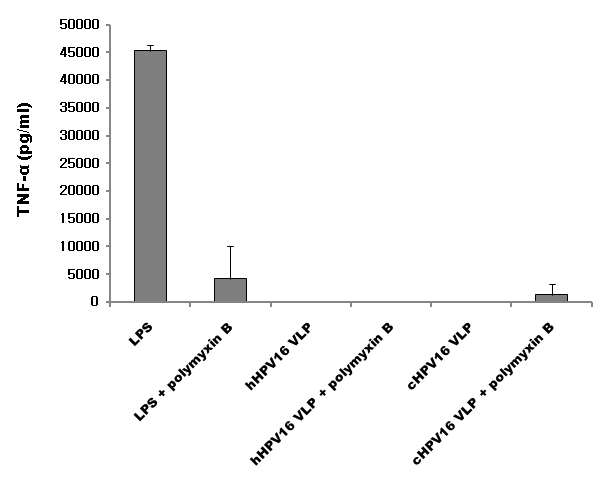

Supplement: Figure S2 — Measurements of endotoxin levels contained in hHPV16 VLP and cHPV16 VLP. To measure the endotoxin levels, RAW264.7 (2×10^4^ cells / well) cells were seeded in a 96-well cell culture plate 24 hours prior to stimulations. To block Toll-like receptor 4 on RAW264.7 cells, the cells were treated with 50 µg/ml of polymyxin B (Sigma, USA) for 30 min at 37°C prior to LPS and HPV VLPs treatments. The cells were treated with LPS, hHPV16 VLP and cHPV16 VLP at concentrations of 10 ng/ml, 10 µg/ml and 10 µg/ml, respectively. Four hours after the treatments, the levels of TNF-α in the culture supernatants was measured using an ELISA kit according to manufacturer's instructions (BD Bioscience, USA). Values are presented as the means ± SD of duplicate assays. (TIF) [file pone.0035893.s002.tif]

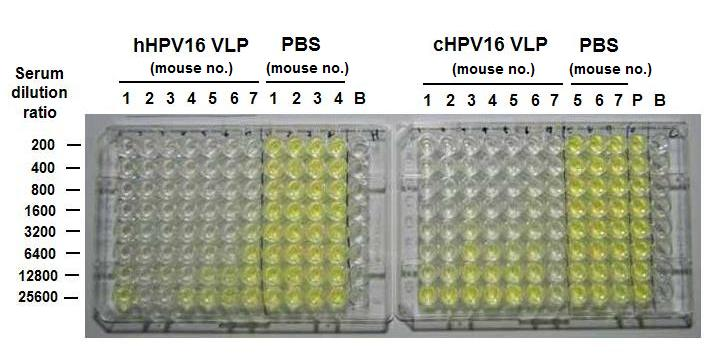

Supplement: Figure S3 — Neutralization assay results. The mice were immunized three times with 1000 ng of hHPV16 VLP or cHPV16 VLP, in combination with aluminum hydroxide (protocol-2, Table 2). The mice sera were serially diluted and incubated with Optiprep density gradient-purified HPV16 PsVs for 1 h at 4°C. The PsV and mice sera mixtures were added to pre-plated 293TT cells and cultured for 72 h at 37°C. The secreted SEAP of each well was developed using a 4-nitrophenly phosphate disodium salt hexahydrate (Sigma, USA). B and P of the figure captions indicate the ells cultured with media only (blank) and PsV only, respectively. (TIF) [file pone.0035893.s003.tif]

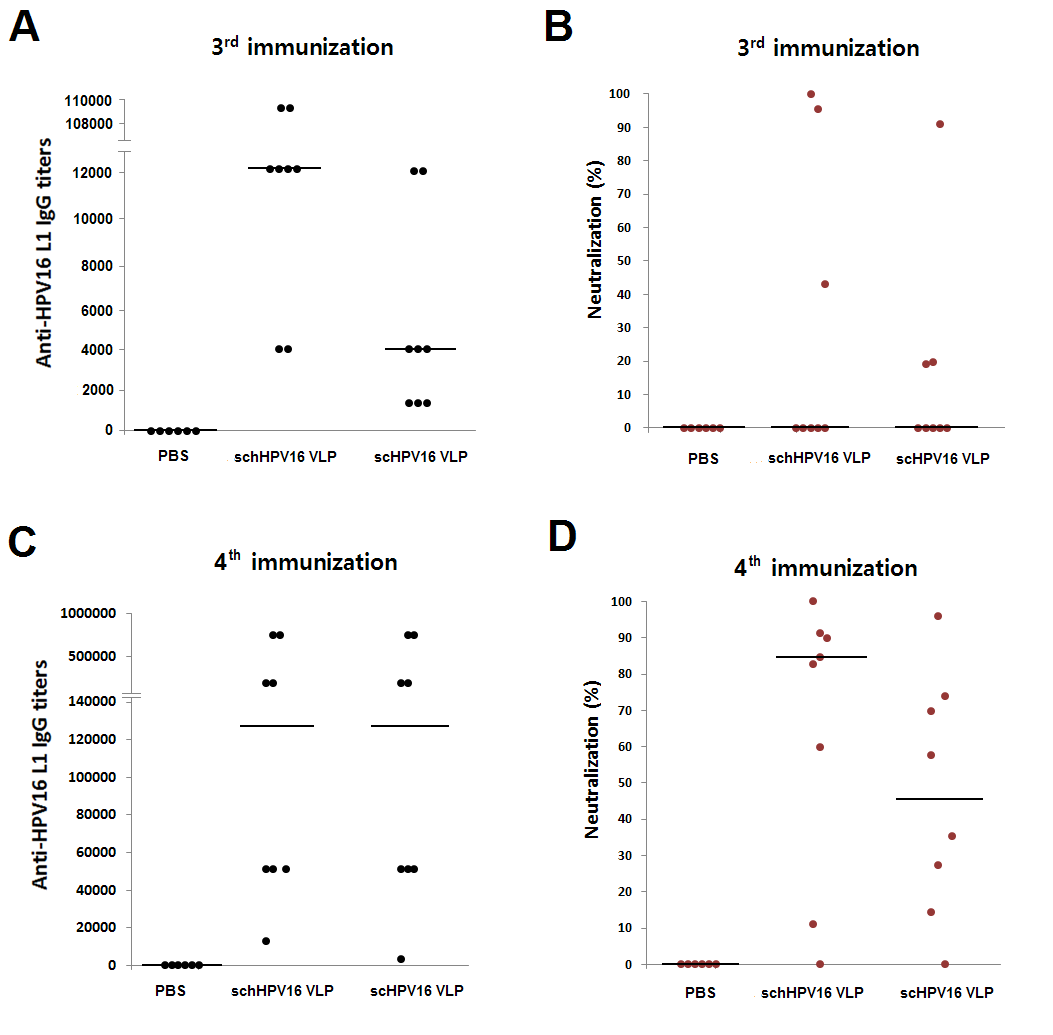

Supplement: Figure S4 — Anti-HPV16 L1 IgG titers and neutralization activities of mice sera following immunizations with schHPV16 and scHPV16 VLP. Mice were immunized subcutaneously three times with 8 ng of schHPV16 VLP or scHPV16 VLP without adjuvant. Ten days after the last immunization, the sera were obtained and analyzed as described in the Materials and Methods to determine the anti-HPV16 L1 IgG titers and neutralization activities. The horizontal bars are the median values (PBS, n = 6; schHPV16 VLP, n = 8; scHPV16 VLP, n = 8). (TIF) [file pone.0035893.s004.tif]
