# Supplementary material for: The Choice of Resin-Bound Ligand Affects the Structure and Immunogenicity of Column-Purified Human Papillomavirus Type 16 Virus-Like Particles
Source: PLoS One. 2012 Apr 26;7(4):e35893. doi: 10.1371/journal.pone.0035893 (PMC3338541; doi:10.1371/journal.pone.0035893)
Supplement: Table S1 — Determinations of LPS levels in Mock and HPV16 VLPs. Mock samples were prepared form the cell lysate of parent cell of HPV16 L1-producing S. cerevisiae. The concentrations of hHPV16 VLP and cHPV16 VLP were determined by SDS-PAGE and Western blotting prior to the assay. The LPS level of each sample was determined using the limulus amoebocyte lysate (LAL) based colorimetric system (ToxinSensor™, GenScript, USA) according to the manufacture's instruction. (DOCX) [file pone.0035893.s005.docx]

| Cell line | Purification method | Sample name | HPV16 L1 (µg/ml) | LPS (EU/ml) | LPS per µg L1  (EU/µg L1) |
| --- | --- | --- | --- | --- | --- |
| *S. cerevisiae* | Heparin chromatography  (method 1 in Table 1) | h-Mock | – | 45 | – |
|  | Cation-exchange chromatography  (method 2 in Table 1) | c-Mock | – | 68 | – |
| HPV16 L1 producing-  *S. cerevisiae* | Heparin chromatography  (method 1 in Table 1) | hHPV16 VLP | 1500 | 740 | 0.49 |
|  | Cation-exchange chromatography  (method 2 in Table 1) | cHPV16 VLP | 1300 | 410 | 0.32 |

Table S1. Determinations of LPS levels in Mock and HPV16 VLPs.

Mock samples were prepared form the cell lysate of parent cell of HPV16 L1-producing *S. cerevisiae*. The concentrations of hHPV16 VLP and cHPV16 VLP were determined by SDS-PAGE and Western blotting prior to the assay. The LPS level of each sample was determined using the limulus amoebocyte lysate (LAL) based colorimetric system (ToxinSensor^TM^, GenScript, USA) according to the manufacture’s instruction.
